# A Personal Financial Simulation for Japanese Hospital-Employed Physicians

**DOI:** 10.7759/cureus.77048

**Published:** 2025-01-06

**Authors:** Yosuke Takakusagi

**Affiliations:** 1 Radiation Oncology, Yokohama Sakae Kyosai Hospital, Yokohama, JPN

**Keywords:** financial asset, financial simulation, hospital employed physician, nisa, personal finance

## Abstract

Introduction

Physicians in Japan are generally high earners, but many face challenges in accumulating sufficient financial assets due to a lack of financial education. This study aimed to examine the savings and investment strategies necessary for hospital-employed physicians in Japan to reach affluence.

Materials and methods

A financial simulation was conducted using average income data for hospital-employed physicians, considering different saving rates (5-40%) and annual investment returns (1-10%). The study also assessed the tax-saving effect of the Nippon Individual Savings Account (NISA).

Results

Hospital-employed physicians can accumulate 100 million Japanese yen in financial assets by saving at least 10% of their income with an annual return of 4% over 40 years. The use of NISA can further reduce taxes, increasing overall returns.

Conclusions

By adopting appropriate savings and investment strategies, including tax-advantaged options like NISA, hospital-employed physicians in Japan can build sufficient wealth for financial security.

## Introduction

Physicians are generally considered to earn a high income. For example, the average annual income of hospital-employed physicians in Japan exceeds three times that of salaried employees nationwide [1,2]; however, many physicians have inadequate financial assets [3]. This issue extends beyond Japan, with reports indicating that physicians in the US also experience economic stress [4]. In both the US and Canada, medical students’ educational debt has become a significant problem [5,6]. Furthermore, many physicians lack basic financial knowledge [7], as medical education does not prioritize financial instruction [5].

In the US and Canada, reports have revealed financial literacy education targeted at medical residents [4,5,7], showing that such education improved residents’ financial literacy. Physicians with inadequate financial literacy may lack proper decision-making criteria when making financial decisions, potentially exposing themselves and their families to excessive risks. Furthermore, low financial literacy can negatively impact well-being and work performance [8]; however, no reports have investigated financial education programs for physicians in Japan.

Securing qualified instructors is the biggest challenge in implementing financial education [7]. In contrast, personal financial simulations do not require instructors; however, no research has examined personal financial simulations for hospital-employed physicians in Japan. In Japan, individuals are considered affluent when their financial assets exceed 100 million Japanese yen (JPY) (approximately 666,700 USD at an exchange rate of 1 USD = 150 JPY) [9]. This study aimed to clarify the required savings rate and investment efficiency for hospital-employed physicians to reach affluence. Furthermore, this study also examines the tax-saving effects of the Nippon Individual Savings Account (NISA).

## Materials and methods

Income calculation for hospital-employed physicians

The income amounts for hospital-employed physicians in Japan (by generation) were calculated based on a survey conducted by the Japan Institute for Labor Policy and Training [10]. The survey was conducted in 2012, targeting hospital-employed physicians, with 3,528 respondents. In this study, income data were derived from Table 5-18 (“Annual Income at Primary Workplaces”) and Table 5-16 (“Annual Income from Multiple Workplaces”). The income for each generation was calculated by adding the average annual income from secondary or additional work locations to the primary employer’s annual income. Only the income from the main employer was considered during the two years of initial clinical training.

Asset simulation

The saving rate was set at intervals of 5%, ranging from 5% to 40%, and all savings were invested as financial assets. The annual rate of return on investments was set between 1% and 10% at intervals of 1%. The years it would take for the financial assets to reach 100 million JPY (666,700 USD) were calculated for each saving rate and annual return. The maximum working period was set at 40 years (from age 25 to age 64).

The following formula was used to calculate the total asset value in the simulation:

$FV=\sum_{n=1}^{N}Cn&middot;(1+r)^{N-n}$,

where FV represents the future value, N is the investment period, Cn is the investment amount for a particular year, and r is the annual rate of return [11]. All asset simulations were performed using Excel for Mac ver. 16.37 (Microsoft Corporation, Redmond, WA, US).

Tax-saving effect of the NISA

In Japan, earnings from financial assets are subject to a tax of 20.315% [12]. In contrast, NISA is a Japanese tax-free investment system designed to encourage personal savings and investments. It provides exemptions from taxes on capital gains and dividends within set annual investment limits. Under the NISA, earnings on up to a cumulative total of 18 million JPY (120,000 USD) of principal (3.6 million JPY per year) are exempt from taxes [13]. The earnings generated by the cumulative principal of 18 million JPY (120,000 USD) were calculated using the above mentioned formula in each simulation. The investment period was 40 years, with an annual rate of return of 4%.

## Results

Income of hospital-employed physicians

Table 1 summarizes the annual income of hospital-employed physicians, showing that the average annual income from secondary sources was 2.19 million JPY (14,600 USD). By age group, the total annual income of hospital-employed physicians included 8.18 million JPY (54,500 USD) for those in their 20s, 11.87 million JPY (79,100 USD) for those in their 30s, 15.46 million JPY (103,100 USD) for those in their 40s, 17.47 million JPY (116,500 USD) for those in their 50s, and 17.06 million JPY (113,700 USD) for individuals 60 and above.

**Table 1:** Annual income of hospital-employed physicians JPY, Japanese yen

Age (years)	Income from primary workplace (million JPY)	Income from another workplace (million JPY)	Total income (million JPY)
20-29	5.99	2.19	8.18
30-39	9.68	2.19	11.87
40-49	13.27	2.19	15.46
50-59	15.28	2.19	17.47
60 and above	14.87	2.19	17.06

Time to reach 100 million JPY in financial assets

**Figure 1:**
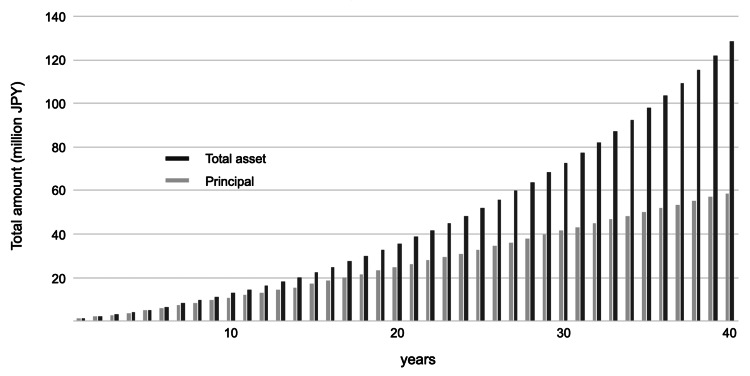
Asset simulation results with a 10% saving rate and a 4% annual return The black bars represent the total asset value (including both principal and returns), while the gray bars indicate the principal amount. This figure illustrates the growth of financial assets over time based on the given saving rate and annual return. JPY, Japanese yen

Table 2 presents the time required to reach 100 million JPY (666,700 USD) in financial assets based on different saving rates and annual returns. When the savings rate was 5%, financial assets did not reach 100 million JPY (666,700 USD) unless the annual return was 7% or higher. At a savings rate of 10%, financial assets did not reach 100 million JPY (666,700 USD) unless the annual return was at least 4%. Under all other conditions, financial assets reached 100 million JPY (666,700 USD) in every simulation. Figure 1 shows the simulation results with a 10% saving rate and a 4% annual return.

**Table 2:** Time required to reach 100 million JPY in financial assets based on different saving rates and annual returns JPY, Japanese yen

Annual return (%)	Saving rate							
	5% (years)	10% (years)	15% (years)	20% (years)	25% (years)	30% (years)	35% (years)	40% (years)
1	NA	NA	40	33	28	25	22	20
2	NA	NA	36	30	26	23	21	19
3	NA	NA	33	28	24	22	20	18
4	NA	37	30	26	23	21	19	18
5	NA	34	28	24	22	20	18	17
6	NA	32	26	23	21	19	17	16
7	38	30	25	22	20	18	17	16
8	36	28	24	21	19	17	17	15
9	34	26	23	20	18	17	16	15
10	32	25	22	19	17	16	15	14

Tax reduction with the NISA

Table 3 summarizes the tax reduction based on different saving rates with an annual return of 4%. Even with the lowest saving rate, the NISA tax reduction amounted to 6.41 million JPY (42,700 USD).

**Table 3:** Tax reduction based on different saving rates with an annual return of 4% JPY, Japanese yen; NISA, Nippon Individual Savings Account

Saving rate (%)	Final amount of investment (million JPY)	Final return (million JPY)	Tax (million JPY)	Tax reduction with NISA (million JPY)
5	63.24	34.76	7.06	6.41
10	126.48	69.53	14.13	8.97
15	189.72	104.29	21.19	10.20
20	252.96	139.06	28.25	10.90
25	316.20	173.82	35.31	11.37
30	379.44	208.58	42.37	11.71
35	442.68	243.35	49.44	11.94
40	505.92	278.11	56.50	12.16

## Discussion

This study performed a personal financial simulation using the average income of hospital-employed physicians. It was suggested that hospital-employed physicians could build sufficient wealth by allocating an appropriate percentage of their incomes to investments.

A survey conducted by the Ministry of Health, Labor and Welfare Japan found that the average annual income of hospital-employed physicians was 14.61 million JPY (97,400 USD) [1]. In contrast, the average income of all salaried workers in Japan was 4.60 million JPY (30,700 USD) [2], indicating that the average income of hospital-employed physicians was 3.18 times higher than the national average. A 2023 public opinion survey on financial behavior conducted by the Financial Services Agency, Japan, found that the average financial assets of households with two or more members were 13.07 million JPY (87,100 USD) [14]; however, a private survey of physicians [3] revealed that 43.4% of households owned less than 10 million JPY (66,700 USD) in financial assets, and 7% had no savings. These surveys suggest that despite relatively high household income, many physicians in Japan cannot accumulate sufficient financial assets. Physicians are highly trusted in society, which may contribute to lower net financial assets due to significant debts such as educational loans and mortgages.

Physicians also face serious financial situations abroad. For example, 40% of residents in the US have no savings, and 10% have credit card debts exceeding 10,000 USD [15]. In a survey of radiation oncology residents in the US, 75% of respondents lacked future financial plans. Poor financial situations can adversely affect personal well-being and job performance [8]; therefore, financial education for physicians is essential. The serious economic issues physicians face are thought to be due to the lack of opportunities for financial education [5]; however, the biggest challenge is securing instructors to provide financial education [7]. Instructors should have sufficient knowledge of asset formation for physicians and be free from conflicts of interest. Nonetheless, while ideally guided by experts, financial simulations (like the one conducted in this study) can also be performed without experts. Financial simulations may improve an individual’s economic situation by identifying saving rates and investment goals.

This study estimated the time it took to reach 100 million JPY (666,700 USD) in financial assets based on saving rates and annual returns from the average income of physicians. According to a 2023 public opinion survey by the Financial Services Agency, the savings rate for households with two or more members was 11% of their income [14]. Considering the survey results, hospital-employed physicians must save and invest at least 10% of their income. The Government Pension Investment Fund (GPIF) manages and operates Japan’s public pension fund. Public pensions require long-term, safe, and efficient asset management, and the GPIF allocates funds evenly among four types of financial assets: Japanese stocks, Japanese government bonds, foreign stocks, and foreign bonds. As a reference for physicians’ investment strategies, the GPIF’s average annual return since 2001 has been 4.26% [16]. Therefore, physicians with limited investment knowledge can expect an annual return of 4% with an investment strategy similar to the GPIF’s. If a hospital-employed physician saves 10% of their annual income and invests it at a 4% annual return for 40 years, their total financial assets will reach 126.48 million JPY (843,200 USD) (Table 3). A saving rate of 10% and an annual return of 4% may be a reasonable initial target for physicians. However, the simulation in this study does not account for inflation. The expected annual return for stock investments, considering inflation, is said to be 4.5% [17]. Taking inflation into account, it may be beneficial to increase the proportion of investments in stocks.

In Japan, a tax of 20.315% is levied on investment returns [11]. Applying a savings rate of 10% and an annual return of 4% for 40 years leaves a final return of 69.53 million JPY (463,500 USD) (Table 3). The tax on selling the financial assets in a lump sum is 14.13 million JPY (94,200 USD); however, with NISA, up to 18 million JPY (120,000 USD) of the principal is exempt from taxes. The tax amount can be reduced by 8.97 million JPY (59,800 USD) by maximizing the use of NISA. NISA allows for investment in a wide range of assets, including stocks, bonds, and real estate, and the investment strategy does not change depending on whether NISA is used or not. Therefore, since NISA provides tax benefits on earnings, it is essential to make optimal use of NISA. However, NISA has an investment limit of 18 million JPY (120,000 USD), so it is not possible to build all assets solely through NISA. We should also consider utilizing other investment systems besides NISA. Japan has established an individual-type defined contribution pension plan (iDeCo) [18]. While conditions exist for participation and contribution amounts, the investment return in iDeCo is tax-free; therefore, hospital-employed physicians must use NISA and iDeCo for efficient asset formation.

Limitations

This study has several limitations. One is that the average income data are from 2012, which may not accurately reflect current income levels. However, the average income of hospital-employed physicians in 2015 was 14.51 million JPY (96,700 USD) [19], and in 2023 it was 14.61 million JPY (97,400 USD) [1], showing no significant change. Therefore, it is assumed that the 2012 data still reasonably reflects the current income, with only minor discrepancies. In addition, it is impossible to precisely determine annual savings amounts because the data are provided for average income by decade. Generally, income increases with age; thus, savings for younger generations may have been overestimated. Moreover, income from multiple workplaces is the average for all generations and does not reflect income differences by age. Furthermore, as this study is a simulation, the final asset amount cannot be guaranteed. Even if an appropriate investment strategy is adopted, there is a possibility that the total annual return could be zero or even negative. Additionally, financial education does not necessarily lead to correct financial decisions [11]. Therefore, the importance of financial simulations for physicians and salaried workers in general is unclear. However, in Japan, the Financial Services Agency has been providing financial simulation tools for the general public since 2021, and their significance has been increasingly emphasized [20]. Further consideration is needed regarding the usefulness of financial simulations and financial education.

## Conclusions

This study examined the personal financial simulation for hospital-employed physicians, revealing that they can accumulate sufficient assets through savings and investment. Utilizing tax-advantaged systems like NISA is recommended for more efficient asset accumulation.
